# Myocardial Involvement in Chagas Disease and Insulin Resistance: A Non-Metabolic Model of Cardiomyopathy

**DOI:** 10.5334/gh.793

**Published:** 2020-04-24

**Authors:** Luis E. Echeverría, Lyda Z. Rojas, Luis A. López, Oscar L. Rueda-Ochoa, Sergio Alejandro Gómez-Ochoa, Carlos A. Morillo

**Affiliations:** 1 Public Health and Epidemiological Studies Group, Cardiovascular Foundation of Colombia, Floridablanca, CO; 2 Heart Failure and Heart Transplant Clinic, Fundación Cardiovascular de Colombia, Floridablanca, Santander, CO; 3 Research Group and Development of Nursing Knowledge (GIDCEN-FCV), Research Institute, Cardiovascular Foundation of Colombia, Floridablanca, Santander, CO; 4 Electrocardiography Research Group, Universidad Industrial de Santander, Bucaramanga, CO; 5 Department of Cardiac Sciences, Cumming School of Medicine, University of Calgary, Alberta, CA; 6 Department of Medicine, Cardiology Division, McMaster University, PHRI-HHSC, Hamilton, Ontario, CA

**Keywords:** insulin resistance, heart failure, Chagas Disease, type 2 diabetes mellitus

## Abstract

**Background:**

Heart failure (HF) and type 2 Diabetes Mellitus (T2DM) represent two chronic interrelated conditions accounting for significant morbidity and mortality worldwide. Insulin resistance (IR) has been identified as a risk factor for HF; however, the risk of IR that HF confers has not been well elucidated. The present study aims to analyze the association between myocardial involvement in Chronic Chagas Cardiomyopathy (CCM) and IR, taking advantage of this non-metabolic model of the disease.

**Methods:**

Cross-sectional study performed during the period 2015–2016. Adults with a serological diagnosis of Chagas disease were included, being divided into two groups: CCM and non-CCM. IR was determined by HOMA-IR index. Bivariate analysis and multivariate logistic regression were performed to determine the association between IR as an outcome and CCM as primary exposure.

**Results:**

200 patients were included in the study, with a mean age of 54.7 years and a female predominance (53.5%). Seventy-four (37.0%) patients were found to have IR, with a median HOMA-IR index of 3.9 (Q1 = 3.1; Q3 = 5.1). Multiple metabolic variables were significantly associated with IR. In a model analyzing only individuals with an altered HWI, an evident association between CCM and IR was observed (OR 4.08; 95% CI 1.55–10.73, p = 0.004).

**Conclusion:**

CCM was significantly associated with IR in patients with an altered HWI. The presence of this association in a non-metabolic model of HF (in which the myocardial involvement is expected to be mediated mostly by the parasitic infection) may support the evidence of a direct unidirectional correlation between this last and IR.

## Introduction

Insulin resistance (IR) is recognized as an independent risk factor for heart failure (HF) for more than a century. Multiple epidemiological studies, such as the Framingham Heart Study and the Reykjavik Study, revealed a 2-fold higher risk of incident HF in patients with type 2 Diabetes Mellitus (T2DM) when compared to patients without this risk factor [1,2,3]. T2DM has also been shown to be a predictor of hospitalization and mortality in the context of HF [4]. However, the possibility of HF causing IR was not considered until recent years, as clinical studies identified a high prevalence of T2DM in HF, which varied from 33% of patients hospitalized with HF in the Euroheart Failure Survey II to 45% in the PARADIGM-HF trial. This prevalence is significantly higher than that observed in the general population (4–7%) [5,6].

Additionally, prospective studies have found a higher risk of incident Diabetes Mellitus (DM) in individuals with HF, as in the study of The Osservatorio Geriatrico Regione Campania Group, in which DM was developed in 29% of the individuals with HF compared to 18% matched controls [7,8]. Guglin et al. found a significantly increased risk of a worsening DM status after three or four years after adjusting for multiple confounding variables [9]. This association is supported by multiple pathophysiological mechanisms commonly observed in HF, as the increase in catecholamine levels, which inhibit pancreatic insulin secretion and stimulates hepatic glycogenolysis and gluconeogenesis, and the shift of myocardial metabolic substrate (from fatty acids to glucose) as a result of the low oxygenation state characteristic of HF, which causes a cardiac accumulation of toxic lipids that inhibit insulin signaling [9,10,11].

Nevertheless, to date, all studies have been performed mostly in ischemic or metabolic heart failure patients, a condition that may inFluence the association between HF and IR by adding additional risk factors, an effect that may persist even after performing adequate multivariate analyses [8,9,11,12]. Chronic Chagas Cardiomyopathy (CCM) patients usually tend to have less metabolic comorbidities when compared to other non-CCM HF individuals, as was evidenced in the study of Shen et al., which compared the clinical characteristics and outcomes in patients with HF with reduced ejection fraction caused by Chagas disease, with other etiologies, finding a significantly lower prevalence of diabetes mellitus, hypertension and myocardial infarction in CCM patients [13]. Moreover, the pathophysiology of the myocardial involvement in CCM is mainly inFlammatory, postulating CCM as a potential unique model to evaluate the effect of HF in determining the risk of IR and DM [14,15,16]. This study aimed to estimate the prevalence of IR and the incidence of DM (defined as the DM that was unknown for the patients until diagnosed during this study) in Chagas Disease and its association with CCM diagnosis in a non-metabolic model of heart failure.

## Methods

### Study Design and Population

A cross-sectional study conducted between January 2016 to January 2017 in the Heart Failure and Transplant Clinic of Foundation of Colombia, Floridablanca, Colombia. Patients older than 18 years with positive IgG antibodies for T. cruzi by indirect immunoFluorescence, ELISA, or haemagglutination test were included. All individuals with diseases associated with cardiometabolic factors relevant to the study such as uncontrolled hypertension, known diabetes mellitus, history of coronary disease or myocardial infarction, valvular disease, Cushing’s disease, pheochromocytoma, acromegaly, polycystic ovary and individuals receiving drugs with hypoglycemic effect (except cardioselective beta-blockers) were excluded.

### Main Exposure

CCM was considered as the primary exposure. For the assessment of its impact in IR included patients were divided into two groups: the first one comprised individuals with the indeterminate form of the disease (serological diagnosis without any electrocardiographic or echocardiographic abnormality) while the second consisted of patients in which a diagnosis of CCM had been previously established associated with typical echocardiographic (segmental or global wall motion abnormalities, cardiac aneurysms) or electrocardiographic (left anterior fascicular block, left bundle branch block, right bundle branch block, atrioventricular blocks, ventricular premature beats, atrial fibrillation or Flutter, bradycardia ≤50 beats/min) alterations consistent with CCM regardless of New York Heart Association (NYHA) class.

### Outcome Measures

IR was defined as the primary outcome. We chose to use the HOMA-IR index (Homeostasis Model Assessment) to determine IR due to its good correlation with the hyperinsulinemic-euglycemic glucose clamp and its technical advantage of requiring only a single plasma sample assayed for insulin and glucose [17]. The Matthews formula was used to calculate HOMA-IR: HOMA-IR = fasting serum insulin (U/l) × fasting serum glucose (mg/dl)/405. A patient was considered to have IR if the HOMA-IR index was ≥2.5. Secondary outcomes were conditions derived from IR, such as prediabetes and established DM diagnosis, the diagnosis of these two conditions was performed according to American Diabetes Society criteria: Prediabetes: Fasting blood glucose 100–125 mg/dL or glycated hemoglobin (HbA1c 6.0–6.4%), DM2: Fasting blood glucose >=126 mg/dL or glycated hemoglobin (HbA1c equal or greater than 6.5%).

### Confounding Variables

As IR is considered a condition with multifactorial causes, we aimed to analyze all the potential variables that could inFluence its development. For this purpose, sociodemographic characteristics (age and sex), relevant past medical history (alcohol consumption), anthropometric measures (including hip-waist index), physical activity (estimated using the International Physical Activity Questionnaires [IPAQ] by a telephonic interview following the World Health Organization recommendations), mean arterial pressure and biological markers, such as total cholesterol and C-reactive protein were measured.

### Statistical Analysis

Continuous distributed normal variables are reported as mean ± SD, non-normally distributed as median with first and third quartiles, and categorical variables are presented as absolute values and percentages. To determine whether there were differences in population sociodemographic and clinical characteristics by insulin resistance, we used Chi-square and fischer exact test for categorical variables and the unpaired Student t or Mann-Whitney U test for continuous variables.

We examined the association of CCM with insulin resistance (HOMA-IR ≥2.5 versus <2.5). We performed a literature search for evaluating the relevant variables to include in the model, along with the ones that could have a potential interaction. We found that in several studies that performed multivariable models considering IR and DM as the outcome measure, hip waist index (HWI) was observed to have a significant p-value for interaction with multiple variables in the models created; therefore, we analyzed HWI as an interaction variable in our analysis [18,19,20]. Multivariable logistic regression models adjusted by sex, age, mean arterial pressure, alcohol, HWI, group*HWI interaction (p-value = 0.086, considering a significant p-value for interaction as p < 0.15), total cholesterol, reactive protein C (CRP) and physical activity, were performed. Given the significant p-value for HWI interaction, we performed a stratified analysis by HWI alteration (Defining a normal HWI as 0.90 or less for men, and 0.80 or less for women). A p-value <0.05 was considered significant. All statistical tests were two-sided. All data were analyzed using Stata Statistical Software, version 14.

The Ethical Review Board of the FCV approved this study. All participants signed an informed consent before their inclusion in the study. This research followed the guidelines stated in the Declaration of Helsinki of the World Medical Association and the Resolution 8430 of 1993 of the Ministry of Health of Colombia.

## Results

**Figure 1:**
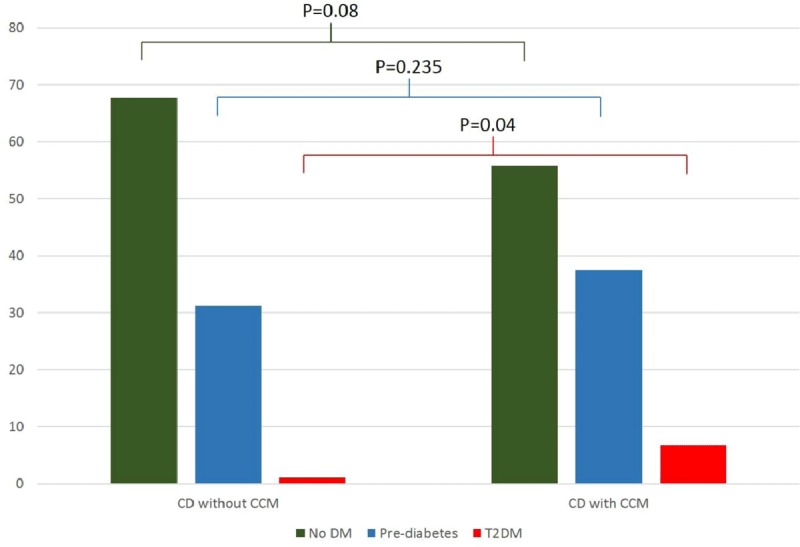
Percentages of Newly Diagnosed DM and Pre-diabetes in Individuals with Chagas Disease with and without Chronic Chagas Cardiomyopathy. **Abbreviations: T2DM =** type-2 Diabetes Mellitus; **CCM =** Chagas cardiomyopathy.

Two hundred patients were included in the study; the mean age was 54.7 years, with 54% being female. Patients in the CCM group (n = 104; 52%) were significantly older (59 ± 11.1 vs 49 ± 11.4 years) and predominantly male (57.69% vs 34.38%) compared to the CD patients without CCM (n = 96; 48%). The overall prevalence of IR was 37% (n = 74), with a median HOMA-IR index of 3.9 (Q1 = 3.1; Q3 = 5.1); both the continuous value and the proportion of patients with IR (HOMA-IR value >2.5) were not significantly different in the CCM group compared to the one without CCM (See supplementary Table 1). No significant differences in age, sex, sociodemographic characteristics, left ventricular ejection fraction (LVEF), NT-proBNP and pharmacological treatment were observed when compared to individuals without IR (Table 1). Body mass index, HWI, systolic, diastolic and mean arterial pressure, C-reactive protein, triglycerides, total cholesterol, and HDL were significantly associated with IR in the bivariate analysis. Interestingly, we found an important prevalence of prediabetes in the studied population (n = 69; 34.5%), also being higher in the group with CCM when compared to those without CCM (37.5% vs. 31.2%; p = 0.235); however, this difference was not statistically significant. finally, the incidence of DM was 4.02% in the overall population, while it was 1.05% in the CD patients without CCM and 6.73% in those with HF (p = 0.04) (Figure 1).

**Table 1:** Bivariate Analysis of the Variables Associated with Insulin Resistance in a Population of Patients with Chagas Disease.

Variable	Insulin resistance (Yes) n = 74	Insulin resistance (No) n = 126	p-value

**Sex**			
Females	46 (62.16)	61 (48.41)	0.060
Males	28 (37.84)	65 (51.59)	
**Age (Years)**	54.9 ± 10.3	54.4 ± 13.3	0.792
**Education**			
None	13 (17.57)	10 (7.94)	0.298*
Incomplete Primary School	28 (37.84)	53 (42.06)	
Complete Primary School	18 (24.32)	36 (28.57)	
Incomplete Secondary School	2 (2.70)	5 (3.97)	
Complete Secondary School	8 (10.81)	17 (13.49)	
Technical	1 (1.35)	1 (0.79)	
Incomplete University	0 (0.00)	2 (1.59)	
Complete University	4 (5.41)	2 (1.59)	
**Occupation**			
None	30 (40.54)	49 (38.89)	0.238
Student	0 (0.00)	3 (2.38)	
Employee	9 (12.16)	5 (3.97)	
Independent	28 (37.84)	53 (42.06)	
Unemployed/Dismissed	5 (6.76)	13 (10.32)	
Pensioner/Retired	2 (2.70)	3 (2.38)	
**Social Security**			
Contributory	25 (33.78)	40 (31.75)	0.677
Subsidized	47 (63.51)	81 (64.29)	
Prepaid	1 (1.35)	0 (0.00)	
Special Regime	1 (1.35)	3 (2.38)	
None	0 (0.00)	2 (1.59)	
**Area of Residence**			
Urban	47 (63.51)	56 (44.44)	0.009
Rural	27 (36.49)	70 (55.56)	
**Smoking**			
No	73 (98.65)	122 (96.83)	0.653
Yes	1 (1.35)	4 (3.17)	
**Alcohol Consumption**			
No	59 (79.73)	90 (71.43)	0.240
Yes	15 (20.27)	36 (28.57)	
**Physical Activity**			
Low	46 (36.51)	34 (45.95)	0.423*
Moderate	31 (24.60)	19 (25.68)	
High	49 (38.89)	21 (28.38)	
**NYHA**			
I	62 (83.78)	97 (76.98)	0.252*
II	10 (13.51)	24 (19.05)	
III	2 (2.70)	5 (3.97)	
**BMI** (**kg/m^2^)**	29.5 (25.9–32.3)	24.1 (21.8–26.8)	0.000
**HWI**	0.93 ± 0.07	0.89 ± 0.08	0.000
**SBP (mmHg)**	125 ± 17	119 ± 15	0.014
**DBP (mmHg)**	76 ± 11	71 ± 9.3	0.000
**MAP (mmHg)**	93 ± 12	87 ± 10	0.000
**LVEF (%)**	56 (45–60)	58 (43–64)	0.479
**Glycaemia (mg/dL)**	99 (95–109)	95 (91–101)	0.000
**Serum Insulin Levels (mIU/L)**	15.3 (12.7–18.9)	6.0 (4.3–7.9)	0.000
**Hb1Ac (%)**	5.5 (5.2–5.7)	5.4 (5.1–5.6)	0.010
**PCR (mg/L)**	2.2 (1.3–4.4)	1.2 (0.6–2.8)	0.000
**Total Cholesterol (mg/dL)**	202 (178–234)	189 (153–215)	0.017
**LDL (mg/dL)**	122 (96–145)	113 (87–136)	0.140
**HDL (mg/dL)**	38 (33–47)	48 (42–55)	0.000
**Triglycerides (mg/dL)**	178 (125–279)	110 (83–151)	0.000
**NT-proBNP (pg/ml)**	101 (42–338)	92 (48–957)	0.236
**Aldosterone (pg/mL)**	53.5 (36.6–77.5)	58.2 (40.7–94.9)	0.181
**Angiotensin-2 (pg/mL)**	27.2 (17.5–39.5)	26.2 (16.5–39.1)	0.581
**Norepinephrine (pg/mL)**	204.1 (95.4–361.1)	233.9 (121.7–404.1)	0.218
**HOMA-IR Index**	3.9 (3.1–5.1)	1.4 (1.0–1.9)	0.000
**ACEI/ARB**			
No	41 (55.41)	79 (62.70)	0.370
Yes	33 (44.59)	47 (37.30)	
**Beta-blockers**			
No	38 (51.35)	77 (61.11)	0.178
Yes	36 (48.65)	49 (38.89)	
**Aldosterone Antagonists**			
No	55 (74.32)	90 (71.43)	0.658
Yes	19 (25.68)	36 (28.57)	
**Diuretics**			
No	59 (79.73)	104 (82.54)	0.621
Yes	15 (20.27)	22 (17.46)	
**Digoxin**			
No	68 (91.89)	119 (94.4)	0.480
Yes	6 (8.11)	7 (5.56)	

This table contains n (%) for categorical variables and median (first and third quartile) or mean (standard deviation) for continuous variables, * Chi2 trend. Abbreviations: BMI: Body Mass Index; HWI: Hip Waist Index ACEI/ARB: Angiotensin-Converting Enzyme Inhibitor/Angiotensin Receptor Blocker.

A multivariate logistic regression model was performed to evaluate the association between CCM and IR accounting for potential confounding variables. An imputation process (Imputation through chained equations) was performed in order to optimize the collected information, as some patients had missing information related to IPAQ questionnaires (18.5%). No variation in the results was observed after the imputation was performed. In the first model, which considered a biological interaction of HWI in the studied association, CCM was not associated with IR; however, when analyzing only individuals with an altered HWI, an evident association was observed (OR 4.08; 95% CI 1.55–10.74, p = 0.004) (Table 2). This could be explained by the fact that the number of individuals with IR and a normal HWI was low (n = 6; 3%), with most patients with IR in the altered HWI group. Sensitivity analysis was performed to evaluate the impact of the imputation process in the results of the model, and no significant differences were observed comparing both models.

**Table 2:** Statistical Models Evaluating the Association between Heart Failure and Insulin Resistance Considering the Potential Confounders.

Insulin resistance	Model 1			Model 2			Model 3		

	OR	95% CI	p-value	OR	95% CI	p-value	OR	95% CI	p-value

CCM (Yes/No)	0.68	0.11–4.31	0.683	**4.08**	**1.55–10.74**	**0.004**	0.38	0.02–6.81	0.516
Sex (Men vs Women)	0.49	0.24–1.01	0.054	0.56	0.26–1.21	0.142	0.11	0.01–1.57	0.103
Age, years (>60 vs ≤60)	0.18	0.07–0.45	0.000	0.19	0.08–0.49	0.001	1	omitted	
MAP, mmHg (>100 vs ≤100)	2.93	1.21–7.11	0.017	2.83	1.12–7.11	0.027	4.34	0.04–465.2	0.538
Alcohol consumption (Yes/No)	0.55	0.24–1.22	0.142	0.54	0.23–1.26	0.155	0.19	0.01–4.44	0.306
HWI (Yes/No)	2.02	0.47–8.66	0.345	—	—	—	—	—	—
Group*HWI interaction	5.94	0.78–44.96	0.084	—	—	—	—	—	—
Total Cholesterol, mg/dl (>200 vs ≤200)	1.61	0.81–3.23	0.175	1.70	0.80–3.61	0.165	0.77	0.04–14.2	0.861
CRP mg/dl (>3 vs ≤3)	1.85	0.87–3.94	0.109	1.59	0.71–3.61	0.260	9.79	0.52–183.7	0.127
Physical activity (Low)									
Moderate	1.38	0.59–3.19	0.452	1.18	0.48–2.87	0.718	8.99	0.48–167.4	0.141
High	1.06	0.41–2.72	0.909	0.98	0.35–2.73	0.976	0.30	0.01–19.53	0.576

Abbreviations: CCM: Chronic Chagas Cardiomyopathy; MAP: Mean arterial pressure; HWI: Hip Waist Index; CRP: Reactive protein C. Model 1 = Analysis with HWI interaction. Model 2 = Including only patients with HWI alteration. Model 3 = Including only patients without HWI alteration.

## Discussion

In this study, adult individuals with a serological diagnosis of Chagas Disease divided into two groups (with and without CCM) were compared to evaluate the association between CCM and IR in a non-metabolic model of heart failure, finding a significant association in individuals with an altered HWI even when adjusting by potential confounding variables. This represents a novel finding of a non-metabolic HF model, such as CCM.

The link between IR/DM and HF was considered to be unidirectional, as the evidence available showed that DM was a significant risk factor both for HF development (8% increased risk of HF with each 1% increase in HbA1c) and for a worse prognosis, increasing the risk of HF admissions and death [2,21]. A bidirectional relationship of this phenomenon has just been recently acknowledged, as epidemiological studies showed a significantly higher prevalence of DM in HF patients. Few studies have addressed the theory of assessing HF as a direct cause of DM [7,8,9,12]. Nichols et al., in a cohort of non-diabetic patients, reported that HF of multiple etiologies was independently associated with a 48% increase in the incidence of DM [12]. Amato and colleagues reported in 1,339 elderly subjects that the diagnosis of HF was associated with a 2-fold increase in the development of T2DM development. Untreated HF correlated with a higher prevalence of DM (4.0%, 95 % CI 3.4–5.8); this association was independent of sex, age, and other confounding variables [7]. Similarly, the severity of HF has been correlated with the degree of insulin resistance, with the latter being higher as NYHA class increases and peak VO2 decreases [22].

**Figure 2:**
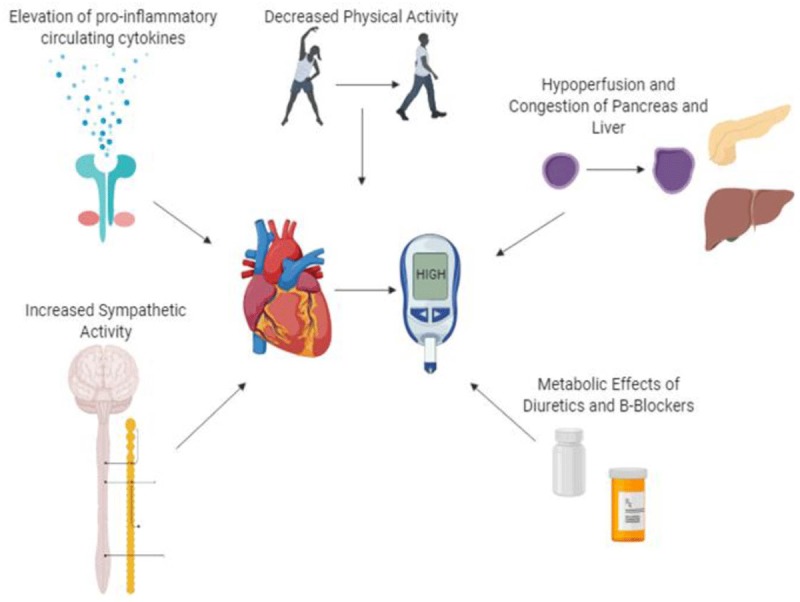
Pathophysiological Pathways for Insulin Resistance in Heart Failure.

The molecular mechanisms underlying this association remain unclear; however, multiple hypotheses have been postulated, highlighting the role of catecholamines, which are typically elevated in HF patients. Such an increase alters cardiac energetic efficiency, favoring lipolysis and elevating the serum levels of free fatty acids, while the increase in sympathetic activity stimulates hepatic gluconeogenesis and glycogenolysis and leads to an inhibited pancreatic insulin secretion, promoting hyperglycemia (Figure 2) [10,11].

HF is characterized by a shift in myocardial metabolism from fatty acids to glucose for ATP production, as obtaining energy from fatty acids requires a higher amount of oxygen available, a condition that is limited under the reduced oxygenation state observed in HF. This shift is relevant because it favors a cardiac accumulation of toxic lipid products, which finally inhibit insulin signaling by reversing the stimulation of protein kinase C activity [23]. Supporting this hypothesis, multiple studies have evaluated the benefit of left ventricular assistance devices (LVAD) in insulin sensitivity in HF patients, favoring an improved blood glucose control after LVAD implantation. Guglin et al. reported a dramatically decreased HbA1C within three months after LVAD implantation that remained at this level for up to 1 year. Similar studies have proved an enhanced myocardial insulin signaling leading to a decrease in insulin resistance, supporting the benefits of LVAD in this aspect [24,25,26]. This benefit may also derive from the resolution of two hemodynamic abnormalities that directly affect the pancreas in HF, namely increased central venous pressure and low cardiac output [27]. Moreover, optimal pharmacological management of HF has known cardioprotective effects and has been shown to alter DM incidence. In the SOLVD and CHARM trials, ACEI/ARB’s demonstrated protective effects against the development of DM [28,29]. However, pharmacological treatment was not associated with IR in the present study.

CCM is characterized by a persistent low-grade T. cruzi infection that has marked systemic effects that also involve metabolic pathways related to insulin sensitivity. This association is partly due to the capacity of the parasite of infecting adipose tissue, leading to a significant infiltration of macrophages into adipose tissue and to chronic inFlammation, disturbing the metabolic processes that are carried out in this specialized tissue [30,31]. These processes are mediated mainly by bioactive mediators known as adipocytokines, being adiponectin the most studied one in the Chagas Disease. Clinical studies in CCM have shown reduced levels of insulin in these individuals, mainly due to an increase in adiponectin levels, simultaneous to a decrease in leptin levels, among other alterations that promote a change in the function of the stimulus-secretion pathway [32,33,34]. Nevertheless, the study of dos Santos et al. reported that among the patients with CD, the individuals with CCM were the only ones with a higher prevalence of T2DM when compared to controls, having the patients with the indeterminate form or the ones with the isolated gastrointestinal form a similar prevalence when compared to healthy volunteers, suggesting that the HF derived from the myocardial involvement of CD may be the primary mechanism of IR in this context [35].

However, even the CCM model is not free of a metabolic inFluence on IR additional to the HF impact by itself; nevertheless, we aimed to mitigate this effect by including CD patients in both groups and evaluating the age in the multivariate analysis as a proxy of the length the parasite had potentially infected the individual. Moreover, the markers of chronic inFlammation (C-reactive protein) and autonomic dysfunction (norepinephrine levels) that could link CD and IR were not statistically different among the two groups, suggesting a more clear and isolated effect of HF diagnosis.

### Study Limitations

As a cross-sectional study, we were not able to perform an analysis of causality between the two conditions studied; therefore, we cannot completely rule out the possibility of reverse causation between IR and CCM. Nevertheless, it is widely known that CCM pathophysiology depends mainly on parasite-dependent damage, immune-mediated tissue injury, neurogenic disturbances, and microvascular derangements [15]; besides, only eight patients had a diagnosis of DM2 in our cohort; Meanwhile, 66 patients had a reduced ejection fraction; Therefore, considering that systolic dysfunction represents a late manifestation of diabetic cardiomyopathy occurring mainly in the long term established DM, the probability of myocardial impairment being attributed to DM is low, making unlikely that IR may be the cause of cardiomyopathy in these patients.

Furthermore, the lack of knowledge of the time of infection of the included individuals avoids performing an accurate analysis of this potentially confounding variable; Nevertheless, the age of the participants may serve as a potential proxy of the time of infection, as it usually occurs during childhood, progressing into cardiomyopathy most commonly after 10 to even 30 years. Patients with a longstanding T. cruzi infection may have lower insulin sensitivity compared to individuals with a more recent onset of the disease. Moreover, the information regarding physical activity was obtained by phone contact after the initial interview, opening the possibility of potential differences with the IPAQ measurement at this initial contact.

## Conclusion

A higher prevalence of IR and DM in this Chagas Disease cohort compared to the epidemiological data of the general population was observed, both in individuals with and without CCM, highlighting the role of T. cruzi infection in glucose regulation mechanisms. A significant association was observed between CCM diagnosis and IR; this relation remained significant after adjustment for sex, age, MAP, alcohol consumption, total cholesterol, CRP mg/dl, and physical activity, considering the CCM*HWI interaction. These findings suggest that in a primarily non-metabolic model of disease such as CCM, HF may play a significant role in the development of IR and subsequent progression to DM.

## Additional File

The additional file for this article can be found as follows:

10.5334/gh.793.s1 Supplementary Table 1. Bivariate Analysis of the Variables Associated with myocardial involvement (CCM) in a Population of Patients with Chagas Disease.

## Abbreviations and Acronyms

**BMI =** Body mass index

**CCM =** Chagas cardiomyopathy

**CD =** Chagas disease

**ECG =** Electrocardiogram

**GLS =** Global longitudinal strain

**HF =** Heart Failure

**HWI =** Hip waist index

**IR =** Insulin Resistance

**LVEF =** Left ventricular ejection fraction

**NT-proBNP =** NT-terminal pro B-type natriuretic peptide

**NYHA =** New York Heart Association

**CRP =** Reactive C Protein

**T2DM =** type-2 Diabetes Mellitus
