# Supplementary material for: Myocardial Involvement in Chagas Disease and Insulin Resistance: A Non-Metabolic Model of Cardiomyopathy
Source: Glob Heart. 2020 Apr 24;15(1):36. doi: 10.5334/gh.793 (PMC7218788; doi:10.5334/gh.793)
Supplement: Supplementary Table 1. — Bivariate Analysis of the Variables Associated with myocardial involvement (CCM) in a Population of Patients with Chagas Disease. [file gh-15-1-793-s1.pdf]

Supplementary File

Supplementary Table 1. Bivariate Analysis of the Variables Associated with myocardial involvement (CCM) in a Population of Patients with Chagas Disease

Variable	Indeterminate patients (n=96)	CCM patients (n=104)	p-value
**Sex**			
Females	63 (65.63)	44 (42.31)	**0.001**
Males	33 (34.38)	60 (57.69)	
**Age (years)**	49.5±11.4	59.2±11.1	**<0.001**
**NYHA**			
I	91 (94.79)	68 (65.38)	**<0.001**
II	5 (5.21)	29 (27.88)	
III	0 (0)	7 (6.73)	
**BMI (kg/m^2)**	26.2 (23.5-29.6)	25.1 (22.4-28.9)	0.288
**HWI**	0.89±0.07	0.91±0.08	0.074
**SBP (mmHg)**	125±13.5	118±17.4	**0.001**
**DBP (mmHg)**	75.3±9.1	71±10.6	**0.002**
**MAP (mmHg)**	91.9±9.7	86.8±9.7	**0.009**
**LVEF (%)**	62 (59-65)	44.5 (33-53)	**<0.001**
**Glycaemia (mg/dL)**	95.3 (91.9-101.6)	97.5 (92.8-104.2)	0.067
**Serum Insulin Levels (mIU/L)**	8.2 (5.4-12.8)	8.3 (5.6-14.1)	0.971
**Hb1Ac (%)**	5.4 (5.2-5.6)	5.4 (5.1-5.7)	0.821
**PCR (mg/L)**	1.4 (0.8-2.6)	1.9 (0.7-4.5)	0.084
**Total Cholesterol (mg/dL)**	208 (179-235)	181 (145-206)	**<0.001**
**LDL (mg/dL)**	126.5 (100-156)	106 (80-130)	**<0.001**
**HDL (mg/dL)**	46.7 (38-53)	43.1 (35.7-52.8)	0.114
**Triglycerides (mg/dL)**	137.9 (96-206)	118 (88-178)	0.182
**NT-proBNP (pg/ml)**	56.8 (30.6-87.4)	481 (132-2019)	**<0.001**
**Aldosterone (pg/mL)**	54.8 (40.5-67.1)	67.8 (40.1-103.3)	**0.008**
**Angiotensin-2 (pg/mL)**	21.2 (15.6-38.3)	30.3 (19.1-42.4)	0.010
**Norepinephrine (pg/mL)**	237.4 (128.3-396.8)	213.3 (93.9-382.5)	0.335
**HOMA-IR Index**	1.98 (1.27-3.11)	2.01 (1.30-3.49)	0.741
**Insulinresistance (HOMA-IR >2.5) (Yes)**	33 (34.38)	41 (39.42)	0.460
**ACEI/ARB**			
No	91 (94.79)	29 (27.88)	**<0.001**
Yes	5 (5.21)	75 (72.12)	
**Beta-blockers**			
No	89 (92.71)	26 (25)	**<0.001**
Yes	7 (7.29)	78 (75)	
**Aldosterone Antagonists**			

No	96 (100)	49 (47.12)	**<0.001**
Yes	0 (0)	55 (52.88)	
**Diuretics**			
No	94 (97.92)	69 (66.35)	**<0.001**
Yes	2 (2.08)	35 (33.65)	
**Digoxin**			
No	96 (100)	91 (87.50)	**<0.001**
Yes	0 (0)	13 (12.50)	
